# Diagnosis and treatment of pulmonary embolism: a multidisciplinary approach

**DOI:** 10.1186/2049-6958-8-75

**Published:** 2013-12-19

**Authors:** Federico Lavorini, Vitantonio Di Bello, Maria Luisa De Rimini, Giovanni Lucignani, Letizia Marconi, Gualtiero Palareti, Raffaele Pesavento, Domenico Prisco, Massimo Santini, Nicola Sverzellati, Antonio Palla, Massimo Pistolesi

**Affiliations:** 1 Department of Experimental and Clinical Medicine, University of Florence, Largo Brambilla 3, Florence 50134, Italy; 2 Cardiac Thoracic and Vascular Department, University of Pisa, Pisa, Italy; 3 Nuclear Medicine Department, AO Ospedali dei Colli – Monaldi, Naples, Italy; 4 Health Sciences and Centre of Molecular and Cellular Imaging (IMAGO), University of Milan, Hospital San Paolo, Milan, Italy; 5 Department of Angiology and Blood Coagulation, University Hospital of Bologna, Bologna, Italy; 6 Department of Cardiothoracic and Vascular Sciences, University of Padua Padua, Italy; 7 Emergency Department, University of Pisa, Pisa, Italy; 8 Department of Clinical Sciences, Section of Diagnostic Imaging, University of Parma, Parma, Italy

**Keywords:** Anticoagulant, Clinical probability, D-dimer, Pulmonary embolism, Venous thromboembolism

## Abstract

The diagnosis of pulmonary embolism (PE) is frequently considered in patients presenting to the emergency department or when hospitalized. Although early treatment is highly effective, PE is underdiagnosed and, therefore, the disease remains a major health problem. Since symptoms and signs are non specific and the consequences of anticoagulant treatment are considerable, objective tests to either establish or refute the diagnosis have become a standard of care. Diagnostic strategy should be based on clinical evaluation of the probability of PE. The accuracy of diagnostic tests for PE are high when the results are concordant with the clinical assessment. Additional testing is necessary when the test results are inconsistent with clinical probability. The present review article represents the consensus-based recommendations of the Interdisciplinary Association for Research in Lung Disease (AIMAR) multidisciplinary Task Force for diagnosis and treatment of PE. The aim of this review is to provide clinicians a practical diagnostic and therapeutic management approach using evidence from the literature.

## Introduction

Pulmonary embolism (PE) is an acute and potentially fatal condition in which embolic material, usually a thrombus originating from one of the deep veins of the legs or pelvis, blocks one or more pulmonary arteries, causing impaired blood flow and increased pressure to the right cardiac ventricle. Pulmonary embolism and deep vein thrombosis are considered to be two manifestations of the same condition, venous thromboembolism, which is the third most common cardiovascular disorder in industrialized countries [1,2]. PE is difficult to diagnose because symptoms are non-specific and clinical presentation of patients with suspected PE varies widely from patients who are asymptomatic to those in cardiogenic shock.

In October 2011 the Interdisciplinary Association for Research in Lung Disease (AIMAR) established a Task Force for diagnosis and treatment of PE with multidisciplinary representation including 3 pulmonologists, 3 internists, 2 emergency care physicians, 1 cardiologist, 1 radiologist and 1 nuclear medicine physician. The members of the Task Force have engaged in interdisciplinary collaborations regarding the diagnostic strategies and treatment of PE. The interdisciplinary organization structure of the present Task Force was designed to support the need of a multidisciplinary approach in the early diagnosis of the disease. The Task Force was asked to structure its recommendations on the diagnosis of PE through a multidisciplinary process that can be dynamically adapted to a rapid changing and increasingly personalized delivery of health care within a structured framework. The Task Force reviewed the literature, and discussed clinical practices in Italy on meetings and conference calls. No attempt was made to grade evidence or recommendations. The present article represents the recommended consensus-based guidelines of the Task Force.

### Epidemiology

Symptomatic venous thromboembolism occurs in 1–2 per 1,000 adults each year, with about a third presenting with PE [1,2]. The incidence of PE correlates strongly with age, being extremely rare in childhood (5 per 100,000 of the population), but increasing exponentially to nearly 500–600 cases per 100,000 in older (> 75 years) age [1-3]. Overall, men and women are affected equally, but women of reproductive age have slightly higher rates of PE because of the association between the disease and pregnancy, and the increased risk conferred by the use of oral contraceptives [1,4]. In older age, the incidence of PE is higher in men than in women [2]. PE-related mortality can be as high as 25% if untreated [2], however, with adequate anticoagulant therapy, this rate decreases to about 2–8% in the 3 months following diagnosis [5,6]. However, the actual figures could be higher than those generally reported because patients who die before diagnosis are usually not included in clinical studies. In the acute phase, i.e. the first month after diagnosis, mortality is influenced by the presence of hemodynamic instability, underlying comorbidities, and immobility [5]. In the long term, i. e. ≥ 1 year after diagnosis, due to comorbidities that are strong predictors of mortality [7] such as malignancy, left-sided congestive heart failure, and chronic lung disease, mortality can reach 24–27%. Malignancy is the most frequent cause of death (35–45%), whereas recurrent PE accounts for 2.5–7.0% [7].

### Risk factors

Pulmonary embolism is currently considered to be the result of an interaction between patient-related and setting-related risk factors. Patient-related predisposing factors are usually permanent, whereas setting-related risk factors are more often temporary. Commonly, more than one risk factor is present, illustrating that PE is a multicausal disease. However, PE can occur in patients without any identifiable predisposing factors.

#### Inherited risk factors

Prothrombotic inherited risk factors are associated with either reduced levels of anticoagulant proteins or increased levels or function of coagulation proteins. In the general population, thrombophilic abnormalities vary in prevalence and also in the risk of PE that they convey. Generally, the overall absolute risk of PE is low, regardless of the increased relative risk caused by the presence of a thrombophilic factor [2]. Deficiencies of natural coagulation inhibitors, such as antithrombin, protein C and protein S, are strong risk factors for PE, but these deficiencies are rare and only account for 1% of all cases of PE. Factor V Leiden and prothrombin (factor II) G20210A are the two more common genetic variants that have been consistently found to be associated with PE, but still only explain a small proportion of PE cases. The search for new genetic variants associated with PE is ongoing and, at the present, the impact of identification of new genetic risk factors on the management of individual patients is unclear. More insight into how genetic risk factors are involved in PE may enable personalized risk profiling in selected patients. However, to be applicable in a clinical setting, the assays must be fast, affordable, and able to detect a combination of clinically relevant genetic factors.

#### Acquired risk factors

Several acquired risk factors for PE have been identified. The highest risk for PE is conferred by surgery (particularly orthopedic surgery, surgery for cancer, and neurosurgery), history of previous venous thronboembolism, immobility for more than 48 h, hospitalization, infection, and cancer [8-14]. In the Prospective Investigative Study of Acute Pulmonary Embolism Diagnosis (PISA-PED), at least one of these risk factors was present in more than 80% of patients with established PE and in about 70% of those without PE [8]. The risk of developing symptomatic PE is 7 fold higher among patients with cancer than in those without cancer and approximately 10% of all PEs are secondary to a known cancer [11]. In a large population-based study, confirmed symptomatic PEs were diagnosed within 2 yrs in 1.6% of 235,149 cancer cases [11], and metastatic disease at the time of diagnosis was the strongest predictor of PE [11]. All haematological and solid tumour types have been associated with PE but the PE risk varies among the various types of cancer. Blom et al. [11] observed the highest risk of PE adjusted for age and sex among patients with haematological malignancies (odds ratio: 28), lung cancer (odds ratio: 22) and gastrointestinal cancer (odds ratio: 20). Adjusting for age, race and stage, diagnosis of PE was a significant predictor of death during the first year for all cancer types [15]. In patients with PE, the prevalence of concomitant cancer, not known before the diagnosis of PE and discovered by routine investigation at the time of PE diagnosis, varies between 4% and 12% [16]. The risk of occult cancer is increased three- to four-fold in patients with idiopathic PE compared with secondary PE [16]. Considering the high incidence of cancer in the initial months following diagnosis PE, screening for an underlying malignancy may be clinically relevant in selected cases.

About two-thirds of PE cases occur during pregnancy and one-third *post partum*. A recent study showed that the risk of PE was increased five-fold during pregnancy and increased 60-fold during the first 3 months following delivery compared with non-pregnant females [17].

Hormone replacement therapy is reported to increase the risk of PE by two- to four-fold [18,19]. However, at variance with the oral route of administration, transdermal oestrogen does not have a first pass effect through the liver and it has been suggested that this might lead to less risk of thrombosis [18,19]. Oral contraceptive therapy increases the risk of PE by two- to five-fold [20]. However, since the absolute risk of PE is low in young females, the annual risk in users of oral contraceptives remains low at two to three cases per 10,000 [20]. The risk is highest during the first year of use. The type of progesteron affects the risk of venous thrombosis, with a two fold higher risk for contraceptives containing a third generation (desogestrel and gestodene) than a second generation (levonorgestrel) progestogen [20].

Other medical disorders associated with increased risk for PE include heart failure, ischemic stroke, acute respiratory failure or intubation, sepsis, acute rheumatic disease, and inflammatory bowel disease [10,13].

### Diagnostic strategies

The diagnostic pathway of PE is guided by two principles. First, accurate and fast identification of patients with PE is critical because PE is a potentially fatal condition and anticoagulation is associated with the risk of major bleeding. A false diagnosis thus exposes patients to unnecessary risk of death from PE or of bleeding which can also be fatal. Second, the use of individual diagnostic tests in isolation may lead to mismanagement of suspected PE. For these reasons, integrated diagnostic approaches that include a combination of different diagnostic tests are preferred. Because use of a validated diagnostic work-up is associated with a substantially diminished risk of complications [21], implementation of such standardized approaches is highly recommended.

#### Clinical probability assessment

In general, the initiating point for any diagnostic approach is the clinical suspicion that should guide the choice of the initial test [22]. Prior to the development of objective testing, the diagnosis of PE was largely based on clinical history and physical examination. Unfortunately, PE cannot be diagnosed or excluded on clinical grounds as symptoms and signs are non-specific [23-25]. However, it has long been recognised that unexplained dyspnoea and/or chest pain are present in about 97% of the patients with proven PE and may be useful to raise the suspicion of PE and to select patients for further diagnostic testing [8]. Therefore, in the diagnostic work-up of PE, the information obtained from the clinical history and a physical examination should be evaluated in conjunction with additional data derived from readily available laboratory tests, such as chest radiography, electrocardiography, and arterial blood gas analysis [26]. The combination of clinical and laboratory data may either increase the clinical suspicion of PE, or suggest alternative diagnoses [26]. Although diagnostic strategies of PE may differ significantly in different clinical contexts and special conditions, the present Task Force recommends that pre-test clinical probability of PE must always be objectively assessed in each patient, while D-dimer measurements should be determined if pre-test probability of pulmonary embolism is low or intermediate. Diagnostic imaging of the chest should be used to assess post-test probability of PE in most patients. Further testing is necessary when the post-test probability of PE is neither sufficiently low nor sufficiently high to permit therapeutic decisions.

#### Pre-test clinical probability of pulmonary embolism

A thorough clinical evaluation is the key step in raising the suspicion of the disease and setting up appropriate diagnostic strategies. A recent study [27] has shown that the vast majority of patients with pulmonary embolism has at least one of four symptoms which, in decreasing order of frequency, are: *a)* sudden onset dyspnoea; *b)* chest pain; *c)* fainting (or syncope); *d)* haemoptysis.

Although the diagnostic yield of individual clinical symptoms, signs and common laboratory tests is limited, the combination of these variables, either by empirical assessment or by a prediction rule, can be used to stratify patients by risk of pulmonary embolism (low, intermediate or high). The results of two broad prospective studies in the 1990s [8,28] indicate that physicians’ estimates of the clinical likelihood of PE, even if based on empirical assessment, do have predictive value. Three objective scoring systems have been tested prospectively and validated in large scale clinical trials: the Wells score [29], the Geneva score [30] and the Pisa score [8]. The three scoring systems perform reasonably well in objectively assessing the clinical probability of PE in outpatients or emergency room patients. The Pisa score [8] seems to perform better than other scoring systems in hospitalized patients [31]. It appears that fully standardized scoring systems, such as the Wells [29] and the Geneva [30] scores, with no implicit evaluation of symptoms (e.g. dyspnoea and chest pain) or simple instrumental findings (e.g. ECG and chest radiograph), did not perform better than subjective clinical judgment of experienced physicians in the PIOPED [28] and the PISA-PED [8] studies. Conversely, interpretation of chest radiographs in patients with suspected PE, as in the Pisa score [8], necessitates a certain level of clinical experience and it is hard to standardize. Whatever scoring method is used, pre-test clinical probability categorizes patients into subgroups with different prevalence of PE, and the positive and negative predictive value of various objective tests is strongly conditioned by the independently assessed pre-test clinical probability [32]. Accordingly, recent international guidelines [33] recommend that the clinical probability of the disease should be assessed in each patient with suspected PE before any further objective testing occurs. Future research is needed to develop standardized models, of varying degrees of complexity, which may find applications in different clinical settings to predict the probability of PE.

#### D-dimer testing

Fibrin D-dimer is a degradation product of cross-linked fibrin, and its levels are elevated in the presence of simultaneous activation of coagulation and fibrinolysis [34]. Consequently, a normal (usually below a threshold of 500 μg/ml) D-dimer level has a high negative predictive value for PE or deep vein thrombosis [34,35]. However, endogenous fibrin production may be increased in a wide variety of conditions including, cancer, inflammation, infection, pregnancy and chronic illnesses [34,35]. Thus, elevated plasma D-dimer levels have a low positive predictive value for PE and deep vein thrombosis [34,35].

The value of D-dimer measurement in the diagnostic work-up of each patient must be considered according to the determined clinical probability of PE and the sensitivity of the particular method of D-dimer measurement employed [34,35]. A negative D-dimer test result, measured by any method, in combination with a low probability clinical assessment, excludes PE with accuracy [34,35]. An intermediate clinical probability also would exclude PE with reasonable certainty if D-dimer is measured by a high-sensitivity ELISA method [35]. It has been shown that the 3-month risk of PE or deep vein thrombosis in untreated patients with a negative D-dimer and a low or intermediate clinical probability is 1% [35]. Conversely, if clinical assessment results in a high probability of PE, a concomitant negative D-dimer test does not exclude PE [35]. The number of patients with suspected PE in whom D-dimer must be measured to exclude one pulmonary embolism episode ranges between three (in the emergency department) and 10 (in hospitalized patients). Therefore, it appears recommendable to consider D-dimer measurement in the diagnostic work-up of pulmonary embolism only in outpatients or in patients in the emergency department with low or intermediate levels of clinical probability. The sensitivity of D-dimer testing for PE increases with the extent of pulmonary embolism [34,35]. D-dimer concentrations are the highest in patients with PE involving the pulmonary trunk and lobar arteries and with perfusion scan defects involving 50% of the pulmonary circulation.

#### Diagnostic imaging of the chest: post-test probability of pulmonary embolism

In recent years, the contribution of computed tomographic angiography (CTA) to the diagnosis of pulmonary embolism has greatly increased as a consequence of the extraordinary advancement in CTA technology. Multidetector CTA, which outlines thrombi in the pulmonary arteries with intravenous contrast medium, has become the most widely used technique for the diagnosis or exclusion of PE, and has almost replaced lung scanning as a screening test and conventional pulmonary angiography as the reference standard for the diagnosis of acute PE [36].

CTA, however, does not escape the simple rule that the combined use of the estimated clinical probability and the results of one noninvasive test substantially increase the accuracy of confirming or ruling out a disease, as compared with either assessment alone. As shown by the PIOPED II trial [37], the predictive value of CTA is high with a concordant clinical assessment, but additional testing is necessary when clinical probability is inconsistent with the imaging results. Several recent studies [38] have shown a positive yield rate of CTA of 10% in patients who are clinically suspected of PE. This may indicate that the wide availability of CTA has led to an overuse of the technique as a screening procedure for pulmonary embolism in the emergency department. It has been suggested that a substantial number of CTAs could be avoided by adhering to the information derived from clinical evaluation and D-dimer testing [38]. Of note, the positive predictive value of CTA varies with the extent of PE, being 97% with main or lobar pulmonary arteries abnormalities , 68% with segmental, but only 25% with isolated subsegmental pulmonary artery abnormalities [39].

Perfusion (Q) lung scanning was introduced 40 years ago as the first chest imaging method for the diagnosis of PE. A normal Q scan excludes pulmonary embolism with great accuracy (i. e. with high sensitivity and high negative predictive value), whatever the pretest clinical probability [33]. However, Q scanning was thought to be poorly specific (low predictive positive value) for PE because common pulmonary diseases such as infections, neoplasms and COPD, can produce decreased blood flow to the affected regions. Ventilation (V) scanning was added to Q scanning to increase the specificity of scintigraphy [28]. This diagnostic approach is based on the flawed expectation that regions of the lung excluded from perfusion by emboli maintain normal ventilation, thus giving rise to V/Q mismatch [28]. This criterion for diagnosing PE is at variance with the notion that ventilation is shifted away from embolized lung regions. The concept that dead space ventilation is not significantly increased in the course of pulmonary embolism was widely held in respiratory pathophysiology before the V/Q scanning approach was developed, as asserted by Comroe [40], who foresaw that *“*decrease in wasted ventilation [ventilation to unperfused or poorly perfused lung] helps the patient but hinders the physician in diagnosis…*”.* This notion is in keeping with the results of the PIOPED trial [28], in which it was shown that a high-probability V/Q scan (Q defects without matching V abnormalities) lacks sensitivity in diagnosing PE, as it fails to identify 59% of pulmonary embolism patients (sensitivity 41%, specificity 97%). The combination of clinical probability and V/Q scan results either confirms or excludes pulmonary embolism in only 30% of patients. The diagnostic value of the Q scan without V imaging was reappraised in the PISA-PED study [8], in which Q scans were read either as compatible with pulmonary embolism when featuring wedge-shaped (segmental) perfusion defects or not compatible with PE when featuring defects other than wedge-shaped or normal perfusion. When compared with the original PIOPED protocol [28], the PISA-PED approach has several advantages: a) Q scanning either confirms or excludes the clinical suspicion of pulmonary embolism, thus virtually eliminating nondiagnostic examinations; b) the sensitivity of lung scintigraphy is greatly increased (86% versus 41%) but with minor reduction of specificity (from 97% to 93%); c) the combination of clinical probability and Q scanning results confirms or excludes pulmonary embolism in about 80% of patients [8].

More recently, the diagnostic performance of Q scanning for pulmonary embolism was confirmed by examining 889 scans from the PIOPED II study [41]. PIOPED II data [37] were used to test the hypothesis that reading Q scans without V scans and categorizing the Q scan as “PE present’, “PE absent” or “Q scan non-diagnostic” can result in clinically useful sensitivity and specificity in a high proportion of patients. The study has confirmed that Q scan and CTA have comparable positive (85 and 86%, respectively) and negative (96 and 95%, respectively) predictive values, with absence of non-diagnostic readings for the Q scan [41]. Accordingly, in 2012 the Society of Nuclear Medicine [33] revised the practice guidelines for lung scintigraphy, reporting that *“*The modified PIOPED II and PISAPED criteria using information from chest radiograph and perfusion scans have been shown to perform equivalently to those including ventilation scintigraphy, with fewer nondiagnostic studies*”.*

#### Diagnostic strategies in different clinical contexts and special conditions

Most clinicians and radiologists feel more comfortable with an anatomical demonstration of whether a clot is present than assessing the probability of pulmonary embolism by looking at V/Q mismatches [28] or evaluating the shape of a perfusion defect [8]. Furthermore, contrary to scintigraphy, in most hospitals, CTA is available 24 h a day, 7 days a week. However, CTA cannot be performed in the whole population of patients suspected of PE. As shown in the PIOPED II trial [37], about 50% of the recruited patients did not undergo CTA because of documented contraindications, such as renal failure, allergy to the contrast agent, possible pregnancy, critical illness, requirement of ventilator support or recent myocardial infarction. In all these conditions, Q scanning could be the preferred alternative approach to the diagnosis of PE. This approach is particularly important for reproductive-age female patients in whom the breast irradiation dose from CTA can be minimized by using the Q scan as the first imaging test. It has been recently shown that contrast medium-induced nephropathy is at least as common as a diagnosis of PE after CTA [42]. Under circumstances in which clinical probability and imaging test (CTA or scintigraphy) results are discordant, further testing, such as lower limb compression ultrasonography, is required to either confirm or exclude the diagnosis. Another practical approach could be to image the pulmonary circulation with CTA if Q scan was the first imaging test used or vice versa.

With the widespread use of CT, particularly for cancer staging, diagnosis of incidental PE has become increasingly common. Incidental PE was reported in about 3% of thoracic scans in one meta-analysis with higher prevalence among hospitalized and cancer patients [43]. Of note, retrospectively many of these patients has symptoms such as fatigue or breathlessness.

### Treatment

#### Risk-adapted therapeutic strategies

Pulmonary embolism represents a spectrum of syndromes ranging from small peripheral emboli causing pleuritic pain to massive PE resulting in cardiogenic shock or cardiac arrest. Indeed, most patients with PE present with normal haemodynamic condition; however, some of them may rapidly deteriorate and manifest systemic hypotension, cardiogenic shock, and sudden death despite therapeutic levels of anticoagulation. Therefore, risk stratification to identify such patients has emerged as a critical component of care.

Severity index scores which allow clinical stratification of patients with PE according to their prognosis have been proposed [44,45]. According with their scores, patients with PE can be assigned to one of the following different classes of risk: *a)* high-risk patients, who represent about 5% of all symptomatic patients, with about a 15% short-term mortality; these patients should be treated aggressively with thrombolytic drugs or surgical or catheter embolectomy [46]; *b)* low-risk patients, with a short-term mortality of about 1%; they might benefit from early discharge or even outpatient treatment [47]; *c)* intermediate risk patients, who represent about 30% of all symptomatic patients and should be admitted to hospital, with potential benefit of thrombolytic treatment. Both low-risk and intermediate risk categories are referred to as non-massive pulmonary embolism [24].

Apart from hemodynamic stabilization and reversal of hypoxemia, the therapeutic goals for acute PE are prevention of appositional thrombus growth, restoration of pulmonary blood flow, and prevention of recurrences. Therefore, in the absence of contraindications, parenteral anticoagulation is mandatory. The therapeutic options available include unfractionated heparin (UFH), low-molecular-weight heparin (LMWH), fondaparinux. When clinical suspicion of PE is high or moderate and testing will not be completed within four hours, initial anticoagulation, with consideration of the bleeding risk, should be initiated before a definitive diagnosis is available [48].

#### Therapeutic strategies in high-risk patients

Patients with confirmed PE and hypotension (i.e. systolic blood pressure <90 mmHg) who do not have a high bleeding risk require immediate systemic thrombolytic therapy because these patients are at an increased risk of death. [48]. Systemic thrombolytic therapy is most commonly used, typically as 100 mg of tissue plasminogen activator given as a two hour infusion [48]. Alternatively, if expertise is available, thrombus removal may be achieved by infusion of lower doses of thrombolytic drug directly into the thrombus, by catheter based fragmentation and aspiration of thrombus, by use of these two modalities together, or by surgical embolectomy (see below). While there is strong evidence from randomized trials that systemic thrombolysis accelerates resolution of PE, its ability to save lives or reduces long term cardiopulmonary impairment remains uncertain [48].

#### Therapeutic strategies in non-high risk patients

Treatment of patients with intermediate and low risk (i.e. non massive embolism) has three phases: the initial phase, the early maintenance phase, and the long-term secondary prevention phase. In these categories of patients anticoagulation using LMWH or fondaparinux in a weight-adjusted dose is considered the cornerstone of the initial treatment [48 also for further references]. The main advantage of LMWH is that they can be administered subcutaneously in fixed weight-adjusted doses without needing monitoring in most cases [49]. The mechanism of action of these heparins is similar to that of unfractionated heparin, but with a more pronounced effect on activated factor X compared with thrombin. The clinical equivalence of LMWH and unfractionated heparin for treating deep vein thrombosis has been confirmed in a meta-analysis [50]. By contrast with unfractionated heparin and LMWH, which are derived from the porcine intestinal tract, fondaparinux is a synthetic compound which is almost identical to the smallest natural component of heparin that can still bind to antithrombin to specifically inhibit activated Factor X. This drug is non-inferior to LMWH and unfractionated heparin in patients with deep vein thrombosis and PE [51,52]. Unfractioned heparin is preferable for patients with a very high risk of bleeding or severe renal insufficiency [51,52].

#### Long-term secondary prophylaxis and prevention of recurrence

For intermediate and low risk patients with PE, international guidelines [48,53] recommend to start administration of vitamin K antagonists (such as warfarin) as early as the first or second day; UFH, LMWH or fondaparinux therapy should be continued in conjunction for at least five days and stopped when the international normalized ratio is in the therapeutic range (2.0 to 3.0) for at least a day [48,53]. Alternatively, LMWH can be continued long term, particularly in patients with cancer associated with PE because of greater compatibility of LMWH with chemotherapy, and difficulty in controlling vitamin k antagonist therapy. Long term LMWH is also used for treatment PE during pregnancy because vitamin k antagonists are teratogenic [48]. For PE secondary to reversible risk factors, treatment with a vitamin K antagonists for three months is recommended. After three months of treatment, patients with unprovoked PE should be evaluated for the risk-benefit ratio of extended therapy. In light of the lasting increase in the risk of recurrence after the initial occurrence of an “idiopathic” PE, we recommend continuing treatment with a vitamin K antagonist for at least three months. Provided anticoagulation is stable and the risk of bleeding is low, indefinite continuation of this therapy should be considered. Patients with PE and malignancies should be treated with LMWH for the first three to six months and they should subsequently receive lifetime anticoagulation with vitamin K antagonists or LMWH.

Recently developed new oral anticoagulants (rivaroxaban and dabigatran), that are directed against factor Xa or thrombin, overcome some limitations of standard therapy, including the need for injection and for dose adjustments on the basis of regular monitoring [48,54,55]. These new anticoagulants are as effective as conventional anticoagulant therapy, and are associated with a lower risk of intracranial bleeding but a higher risk of gastrointestinal bleeding [54,55]. Dabigatran is preceded by initial heparin therapy, whereas rivaroxaban does not require heparin therapy but requires a higher dose for the first three weeks of treatment [54,55]. Both are contraindicated in the presence of severe renal impairment and, due to the limited experience, must be used cautiously to treat PE in patients with advanced cancer or receiving cancer chemotherapy. The decision to use one of these new drugs for the long term treatment of PE is influenced by local availability and licensing, whether their cost is covered by state or private insurance plans, and patient and physician satisfaction with current therapy.

#### Surgical and interventional treatment of pulmonary embolism

For patient with hypotension or shock in whom thrombolysis has failed or is absolutely contraindicated, surgical embolectomy can be a lifesaving treatment option, provided that the surgery can be performed on specialized center [48]. Alternatively, catheter embolectomy or thrombus fragmentation may be considered, provided that there is adequate experience with these modalities on site. Surgical removal of pulmonary emboli is also generally recommended in the case of patients who have free-floating thrombi in the right atrium or ventricle and in the case of those with impending paradoxical emboli through a patent forame ovale [53]. Recent technical advances in transportable extracorporeal assist systems, and particularly the timely involvement of the cardiac surgeon as part of an interdisciplinary approach to high-risk PE may contribute to better postoperative outcomes.

Systematic use of venous filters to prevent PE recurrence is not recommended [48]. Venous filters may, however, be indicated in exceptional cases if therapeutic anticoagulation is absolutely contraindicated or a PE has recurred despite adequate anticoagulation [48]. The venous filter should be removed as soon as possible in order to avoid secondary vena cava thromboses and thromboembolisms.

## Conclusions

Pulmonary embolism is a potentially life-threatening condition, difficult to diagnose in several patients. Misdiagnosis is frequent particularly in elderly patients. Clinical sings of PE are neither sensitive nor specific enough to rule in or out the diagnosis. The choice of a diagnostic strategy for PE depends on the pretest clinical probability of PE, the condition of the patient, the availability of the necessary test, the risks of testing, the risk of an inaccurate positive or negative diagnosis, and the cost. Clinical evaluation makes it possible to classify patients into probability categories corresponding to an increasing prevalence of PE, whether assessed by implicit clinical judgment or by a validated prediction rule. Structured models to assess clinical probability so far developed have different performances in patients of the emergency department and in those who are hospitalized. Exclusion of PE by clinical probability assessment and D-dimer spares the cost and radiation of an imaging evaluation. CTA has become the method of choice for imaging the pulmonary vasculature when pulmonary embolism is suspected in routine clinical practice. Scintigraphy can be considered the preferred alternative chest imaging technique for patients with contraindication to CTA. If scintigraphy is used, eliminating the V scan can reduce cost and radiation load with gain in diagnostic yield.

## Competing interests

All authors are members of the Interdisciplinary Association for Research in Lung Disease (AIMAR) Task Force on diagnosis and treatment of Pulmonary Embolism. They received reimbursement for attending the meetings of the Task Force.
